# Determinants of selenium status in healthy adults

**DOI:** 10.1186/1475-2891-10-75

**Published:** 2011-07-18

**Authors:** Gerald F Combs, Jennifer C Watts, Matthew I Jackson, LuAnn K Johnson, Huawei Zeng, Angela J Scheett, Eric O Uthus, Lutz Schomburg, Antonia Hoeg, Carolin S Hoefig, Cindy D Davis, John A Milner

**Affiliations:** 1 Grand Forks Human Nutrition Research Center, USDA-ARS, Grand Forks, ND, USA; 2 Institut fuer Experimentelle Endokrinologie, Berlin, Germany; 3 Nutritional Sciences Research Group, Nutrition and Cancer Program, National Cancer Institute, Bethesda, MD, USA

## Abstract

**Background:**

Selenium (Se) status in non-deficient subjects is typically assessed by the Se contents of plasma/serum. That pool comprises two functional, specific selenoprotein components and at least one non-functional, non-specific components which respond differently to changes in Se intake. A more informative means of characterizing Se status in non-deficient individuals is needed.

**Methods:**

Multiple biomarkers of Se status (plasma Se, serum selenoprotein P [SEPP1], plasma glutathione peroxidase activity [GPX3], buccal cell Se, urinary Se) were evaluated in relation to selenoprotein genotypes (GPX1, GPX3, SEPP1, SEP15), dietary Se intake, and parameters of single-carbon metabolism in a cohort of healthy, non-Se-deficient men (n = 106) and women (n = 155).

**Conclusions:**

Plasma Se concentration was 142.0 ± 23.5 ng/ml, with GPX3 and serum-derived SEPP1 calculated to comprise 20% and 34%, respectively, of that total. The balance, comprised of non-specific components, accounted for virtually all of the interindividual variation in total plasma Se. Buccal cell Se was associated with age and plasma homocysteine (hCys), but not plasma Se. SEPP1 showed a quadratic relationship with body mass index, peaking at BMI 25-30. Urinary Se was greater in women than men, and was associated with metabolic body weight (kg^0.75^), plasma folate, vitamin B_12 _and hCys (negatively). One *GPX1 *genotype (679T/T) was associated with significantly lower plasma Se levels than other allelic variants. Selenium intake, estimated from food frequency questionnaires, did not predict Se status as indicated by any biomarker. These results show that genotype, methyl-group status and BMI contribute to variation in Se biomarkers in Se-adequate individuals.

## Background

That selenium (Se) plays roles in cancer prevention has been demonstrated in numerous studies with a variety of animal and cellular models [1-4] and in several clinical trials [5-7]. Yet, the largest study, the Selenium and Vitamin E Cancer Prevention Trial (SELECT), failed to detect cancer risk reduction with Se-supplementation [8], indicating that Se-supplementation may not benefit all individuals.

Rayman et al [9] pointed out that the results of SELECT are consistent with those of the Nutritional Prevention of Cancer (NPC) Trial [7,10]. In NPC, reduced prostate cancer risk was observed mostly among subjects with baseline plasma Se concentrations in the lowest tertile of the cohort, < 106 ng/ml [10]; no risk reduction was noted for subjects with plasma Se > 123 ng/ml, concentrations comparable to those of the subjects in SELECT, which averaged 136.5 ng/ml at baseline [8]. Therefore, it is possible that Se-supplementation may yield anti-cancer benefits in individuals who are below some threshold of Se status without being deficient in the nutrient.

Assessment of Se status is difficult in individuals who are not deficient, such as those of NPC and SELECT. It is well established that Se-deficient individuals show subnormal levels of several Se biomarkers, including some with functional significance, such as the selenoproteins, and others that indicate the amounts of Se in the body, such as the Se contents of tissues and body fluids. Non-deficient individuals, however, have maximal selenoenzyme expression [11-13], rendering those parameters non-informative regarding changes in Se intake. Thus, the total Se content of plasma has become the default biomarker of Se status in non-deficient cohorts.

Plasma Se, while easily measured, is not a single entity. It has several components, which, with our current knowledge, are currently defined as: two selenoproteins (selenoprotein P [SEPP1] and the extracellular GPX3), which specifically contain selenocysteine (SeCys) [14,15]; Se incorporated non-specifically as SeMet in lieu of methionine in albumin and other proteins [16,17]; and a small amount of non-protein bound Se [18,19]. These selenoproteins are fully expressed in Se-adequate individuals; they comprised 22% (SEPP1) and 9% (GPX3) of plasma Se in an individual fed SeMet [19]. In contrast, the non-specific incorporation of Se into plasma proteins appears to be regulated only by SeMet supply for which reason this component would be expected to increase in response to supplementation with SeMet [19], as in SELECT [7], or a source of SeMet such as Se-yeast, as in NPC [8].

We determined multiple biomarkers of Se status in the cohort of healthy Americans in order to elucidate the relationships among those biomarkers and biological, metabolic and genetic factors relevant to Se metabolism. Herein we report the results of an extensive assessment of Se status in that cohort.

## Methods

### Subjects

**Figure 1:**
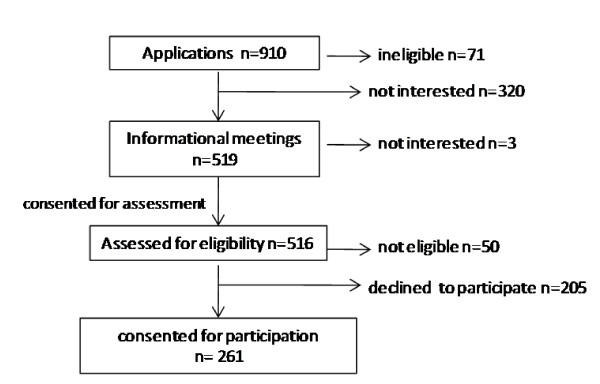
**Selection and randomization of subjects**.

This study involved healthy men and women living in vicinity of Grand Forks, ND, who volunteered and met the following eligibility criteria: over 18 yrs., no chronic liver or kidney diseases (based on blood chemistries and urine analyses), nonsmokers, not having used supplements providing > 50 ug Se/day in the past 6 months (to accommodate users of most OTC multivitamin/mineral supplements), not currently using medications that adversely affect liver or kidney function, body mass index (BMI) ≤ 40 kg/m^2^. A total of 356 volunteers were screened. Of 282 who met the eligibility criteria, 261 subjects (106 men, 155 women) were enrolled in the study; selection and randomization of subjects is shown in figure 1 and subject characteristics are indicated in table 1. Each volunteer was given a cash honorarium pro-rated for the duration of his/her participation in the study.

**Table 1:** Characteristics of the study cohort (N = 261^1^)

characteristic	value
*Anthropometry*	
age, y	49.6 ± 16.3^2^
BMI	27.4 ± 5.0^2^

*biomarkers of Se status*	
plasma Se, ng/mL	142.0 ± 23.5^2^
plasma GPX3, nmoles NADPH/min/mg protein	3.64 ± 0.54^2^
serum SEPP1, μg/mL	3.43 (2.61, 4.51)^3^
buccal cell Se, ng/mg protein	8.39 (5.59, 12.59)^3^
urine Se, ng/mg creatinine	55.5 (40.8, 75.6)^3^

*other metabolic markers*	
serum folate, μg/mL	25.1 (13.2, 47.8)^3^
serum vitamin B_12_, pg/mL	496 (313, 786)^3^
serum homocysteine, μg/mL	6.8 (5.2, 8.9)^3^
TSH, μIU/mL	2.35 (1.21, 4.56)^3^
free T_4_, nmol/mL	1.35 ± 0.18^2^
free T_3_, nmol/mL	2.91 ± 0.59^2^
glucose, mg/dL	89.9 ± 13.1^2^

*dietary intakes*	
energy, kcals/d	2177 ± 721^2^
protein, g/d	87.2 ± 29.4^2^
fat, g/d carbohydrate (g/d)	84.9 ± 33.7^2 ^272.52 ± 104.1^2^
Se, μg/d	109 ± 43^2^

^1 ^155 women, 106 men

^2 ^mean ± S.D.

^3 ^geometric mean (-1 S.D., +1 S.D.)

Oversight was provided by the University of North Dakota Human Subjects Committee which reviewed and approved the protocol. The purposes and procedures of the study were explained to the volunteers verbally and in writing, and written informed consent was obtained from each volunteer.

### Anthropometry

Body weight was measured using an electronic scale. Height was measured using a wall-mounted stadiometer.

### Assessment of Dietary Intake

Dietary intake over the three previous months was assessed by a single, self-administered food frequency questionnaire (FFQ) patterned after the Harvard Service Food Frequency Questionnaire format [20]. This FFQ includes 78 food items without serving sizes indicated (natural portion implied: e.g., one cup of milk, one slice of bread). All food items on the FFQ were matched to food codes from the USDA Nutrient Database for Standard Reference [21] or USDA Food and Nutrient Database for Dietary Studies [22]. For each food item, subjects designated their average consumption by marking one of nine frequency categories ranging from "zero per month" to "six or more times per day". The frequency chosen for each food item was converted to daily intake, e.g., a response of "1-3 per month" was converted to 0.07 servings per day (two servings per month). Total energy, protein, carbohydrate and fat intake were calculated based on nutrient data for the food codes matched to the FFQ food items. Selenium intake cannot be calculated as selenium content of foods varies widely, dependent on crop location. We therefore utilized the Se core foods list developed by Schubert et al [23] to provide suggested food intake patterns. Foods were also sorted into groups using information from the MyPyramid Equivalents Database for USDA Survey Food Codes [24] as well as Friday and Bowman [25].

### Sample Collection and Preparation

Blood was collected by venipuncture in duplicate 7 ml samples into heparinized, EDTA-treated and non-treated glass tubes. Aliquots of whole blood were subjected to low-speed centrifugation to prepare erythrocyte, buffy coat, plasma and serum fractions. Urine (24 hr samples) was collected in sterile polycarbonate bottles. These specimens were held at 4°C pending the completion of screening analyses; excess portions were held at -80°C. Buccal cells were collected using a sterile toothbrush according to Paetau, et al. [26]; cells were lysed in distilled water and lysates were held at -80°C for analysis.

### Clinical Biochemical Analyses

Blood cells were counted using an automated system (CellDyne 3500, Abbott Laboratories, Abbott Park, IL). The activities of aspartate aminotransferase (E.C. 2.6.1.1) and alanine aminotransferase (E.C. 2.6.1.2) were determined in serum using kits (AST2 and ALT2, respectively, JAS Diagnostics, Inc., Miami, FL) and an automated chemistry analyzer (Cobas Mira, Roche Diagnostic Systems, Inc., Sommerville, NJ). The same analyzer was used to determine blood urea nitrogen (BUN) in serum, glucose and protein in plasma, and creatinine in urine (#47381, #0458976160, #44903 and #47003, respectively, Roche Diagnostic Systems, Inc., Sommerville, NJ). Thyroid-stimulating hormone (TSH), non-protein bound thyroxine (T_4_) and triiodothyronine (T_3_) were measured in serum using an automated, solid-phase, two-site chemiluminescent immunometric assays (LKTS1, LKF41 and LKF31, respectively, Immulite 1000 System, Diagnostic Products Corp., Los Angeles, CA).

### Biomarkers of Se Status

Selenium status was assessed on the basis of the activity of GPX and the amount of SEPP1 in serum, and the amounts of Se in plasma, buccal cells and urine. The activity of GPX3 (E.C. 1.11.1.9) was determined in plasma by the method of Paglia and Valentine [27] as modified by Lawrence and Burk [28]. Due to its affinity for heparin, SEPP1 was measured in serum by an enzyme-linked immunoassay [29]. Selenium was determined in plasma, buccal cells and urine by automated electrothermal atomic absorption spectrophotometry using a reduced palladium matrix modifier and an instrument equipped with L'Vov platforms [7]. Certified Standards were used (Alfa Aesar [Ward Hill, MA, USA], Perkin Elmer [Waltham, MA, USA] and CPI [Santa Rosa, CA, USA]) to prepare a calibration set daily with each batch. Continuing calibration certification and a continuing calibration blank were included at 10% frequency and at the beginning and end of the daily batch. An initial calibration verification from an alternate supplier was validated the calibration set at the beginning and end of each analytical series. Matrix effects for plasma and urine were evaluated using quantitative plasma and urine standards (NIST [Gaithersburg, MD, USA], Seronorm [Billingstad, Norway] and Utak [Munich, Germany]) to validate the percentage recovery of the analyte in these sample matrices. As there is no commercially available quantitative standard for Se in buccal cells, matrix effects of buccal cell preparations were accounted for in the analysis by performing spike recoveries using certified calibration standards added directly to one of the samples.

### Other Biochemical Analyses

Folic Acid, B_12 _and homocysteine (hCys) were measured in serum by automated solid-phase, competitive chemiluminescent enzyme immunoassays (LKF01, LKVB1 and LKH01, respectively, Immulite 1000 System, Diagnostic Products Corp., Los Angeles, CA). Vitamin 8-Hydroxy-2'-deoxyguanosine was measured in urine using a competitive enzyme-linked immunosorbent assay (#21026, Bioxytech, Oxis Health Products, Inc., Foster City, CA).

### Genotyping

The genotypes of subjects were determined for genes for several selenoenzymes (two glutathione peroxidases, *GPX1, GPX4; *the transporter selenoprotein P, *SEPP1; and intracellular SEP15*). Genomic DNA was extracted from blood samples using a DNA isolation kit (Qiagen, Valencia, CA). DNA was used as a template for PCR amplification by modifications of previous reported procedures [30-33]. Negative controls were included. Unless otherwise noted, PCR conditions were: 95°C - 3 min; 30 cycles at 95°C - 30 s; 55°C - 60 s; 72°C - 90 s; 72°C - 10 min. Amplified DNA was digested with the appropriate restriction enzymes, and digestion products were separated electrophoretically in 3% agarose unless otherwise noted.

#### SEP15; rs5845 and rs5859

PCR primers [30] 5'-CAGACTTGCGGTTAATTATG-3'and 5'-GCCAAGTATGTATCTGATCC-3' were used to generate a 413-bp amplification product of rs5845 and rs5859. Amplified DNA was digested with Dra I restriction enzyme at position 811, or Bfa I restriction enzyme at position 1125.

#### GPX4; rs713041

PCR primers [31] 5'-GACCTGCCCCACTATTTCTA-3' and 5'-GTCTGTTTATTCCCACAAGG-3' were used to generate 221-bp amplification product of rs713041. PCR conditions were as follows: 94°C - 2 m; 30 cycles at 94°C - 30 s; 57.5°C - 30 s; 72°C - 1 min; 72°C - 7 m. Amplified DNA was digested with restriction enzyme StyI.

#### GPX1; rs1050450

PCR primers [32] 5'-TGTGCCCCTACGCAGGTA-3' and 5'-CCAAATGACAATGACACAGG-3' were used to generate a 337-bp amplification product of rs1050450. Amplified DNA was digested with the Apa I restriction enzyme to identify C/T polymorphism at allele position 679, corresponding to amino acid position 198 of the human GPX1 protein.

#### SEPP1; rs3877899

PCR primers [33] 5'-CACGCATTATTCCTATCTCTATAAGCTTG-3' and 5'-GGAAATGAAATTGTGTCTAGACTAAATTGG-3' were used to generate a 722-bp amplification product of rs3877899. To determine a G/A variant at position 24731 in *SEPP1 *mRNA and a second G/A variant (rs7579) at position 25191 of the reference mRNA sequence in the 3'UTR of mRNA, PCR products were sent for sequencing (Cogenics, Newton, MA) with sequencing primers 5'-CACGCATTATTC-CTATCTCTATAAGCTTG-3', 5'-TCACCTGACA-GTGTAAAGAAAACCTC-3'.

### Statistical Analyses

All statistical analyses (Pearson and Spearman correlations, regression analysis, ANOVA, non-parametric discriminate analysis, Tukey contrast and orthogonal contrast) were performed using SAS Version 9.1.3 (SAS Institute, Inc., Cary NC). Data for buccal Se, urine Se, SEPP1, folate, hCys and vitamin B_12 _were highly skewed and were logarithmically transformed so that their distributions would more closely approximate a normal distribution. For these variables, we report the geometric mean with a 1 SD interval. All other data are expressed as mean ± SD Pearson correlations and linear regressions were computed between biomarkers of selenium status and demographic variables, Se intake, and measured biochemical parameters. Because of the large variability observed in the intakes of the food groups, Spearman correlations were used to assess the relationship between plasma Se and intakes of various food groups. A discriminate analysis was performed to determine whether estimated Se Intake could predict the relative magnitude of plasma Se using the first and fourth quartiles of the latter. A nonparametric method was used because the intake values were not normally distributed. For the SEPP1 and BMI relationship, further analysis was performed by use of both Tukey contrast and orthogonal contrast to validate the correlation.

## Results

### Estimation of Dietary Selenium Intake

**Figure 2:**
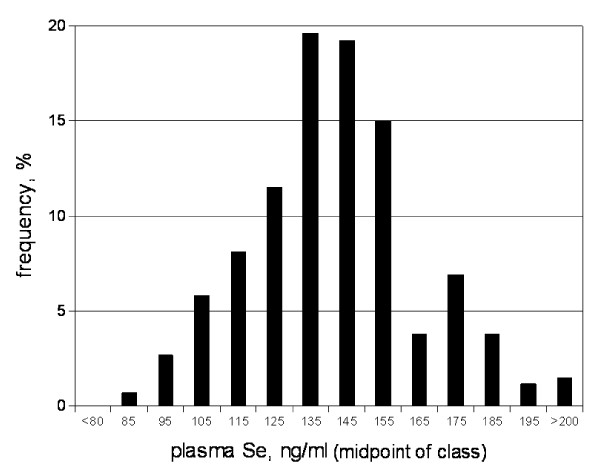
**Frequency distribution of baseline plasma Se levels**.

This cohort was of relatively high Se status, as indicated by plasma Se level (142.0 ± 23.5 ng/ml) (figure 2) other biomarkers of Se status (Table 1) and estimated daily Se intake of 109.1 ± 43.6 μg/d. The major dietary sources of Se were whole wheat bread/rolls, eggs, and spaghetti/other pasta with sauce, which accounted for 21.8% of estimated intake. The estimated Se intakes of men (122 ± 51 μg/d) were highly significantly (p < 0.001) greater than those of women (101 ± 35 μg/d). A core group of 22 foods provided 80% of the estimated amounts of Se consumed by this cohort; 9 of those foods provided half of estimated daily Se intakes. The rankings of these foods as sources of Se were similar for men and women.

### Se Distribution in Blood

The major components of plasma Se were estimated from the total plasma Se and the measured plasma GPX3 activities and serum SEPP1 amounts using the following assumptions; for the amount of SeCys, GPX3 enzyme rate constant of 2.8 × 10^4 ^nmol/min/mg, molecular weight 92 kD, and (stoichiometry of 4 g-atoms Se per mole as SeCYS [34]; for SEPP1 (glycosylated), average molecular weight 60 kD and stoichiometry of 9.9 g-atoms Se per mole as SeCYS [35]. An assumption inherent in the selection of this blood fraction for SEPP1 is that insignificant amounts of SEPP1 protein are removed during the clotting process. We employ this common assumption, with the further caveat that SEPP1 loss by clotting is assumed to be less than the variance of SEPP1 found in the studied population (Coefficient of Variation ~27%).

**Figure 3:**
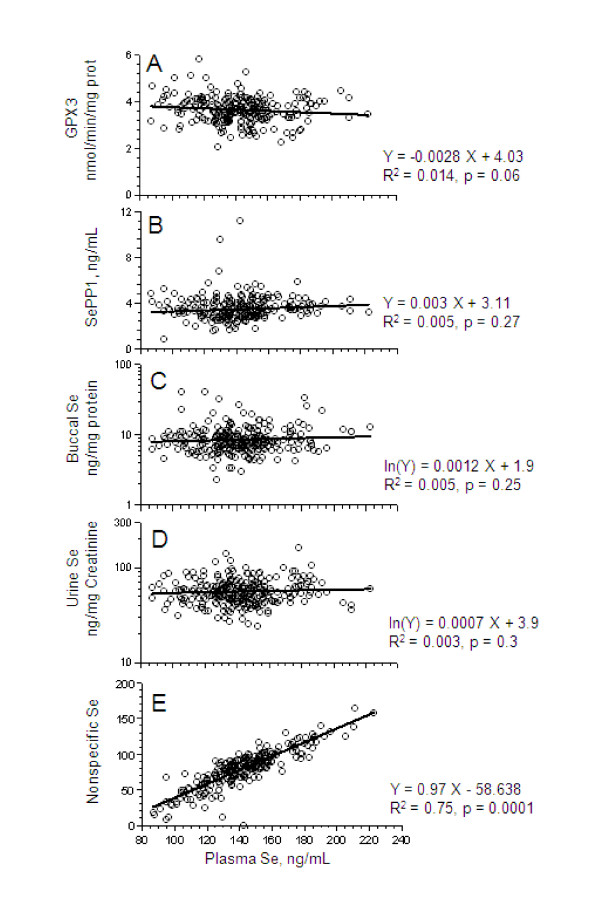
**Relationships of plasma Se level and values of other biomarkers of Se status**. *From top to bottom: *Panel A. Plasma GPX3; Panel B. Serum SEPP1 level; Panel C. Buccal cell Se level; Panel D. Urinary Se level; Panel E. Plasma non-specific Se (as described in the text).

The difference between the total measured Se and the amounts of Se corresponding to the activity of GPX3 and measured amount of SEPP1 was taken as the amount of Se incorporated non-specifically into plasma proteins, presumed to be predominately SeMet. By these estimates, GPX3 and SEPP1 comprised approximately 20% and 34%, respectively of Se, while 47% of Se was present as non-specific components (Table 2). Neither the activity of GPX3, the amount of SEPP1 in serum, nor the amounts of Se in buccal cells or urine were significantly related to total plasma Se concentration. However, the non-specific component of plasma Se was positively associated (r = 0.87, p < 0.0001) with plasma Se (figure 3). SEPP1 concentration was associated with plasma concentration of hCys, a marker of methylation status (r = 0.13, P < 0.05) on an exponential basis.

**Table 2:** Components of blood Se

component	amount of Se ng/ml	% total
Plasma GPX3-SeCYS^1^	27.9 ± 4.3^5^	20.2 ± 5.1^5^
Serum SEPP1-SeCYS^2^	46.5 ± 13.7	33.5 ± 11.6
Total non-specific Se^3^	68.5 ± 27.1	46.5 ± 14.0

total^4^	142.6 ± 24.3	100%

^1^estimated from measured enzyme activity; see text for details.

^2^estimated from measured protein levels; see text for details.

^3^imputed; see text for details.

^4^determined by direct measurement

^5^mean ± S.D., n = 233

**Figure 4:**
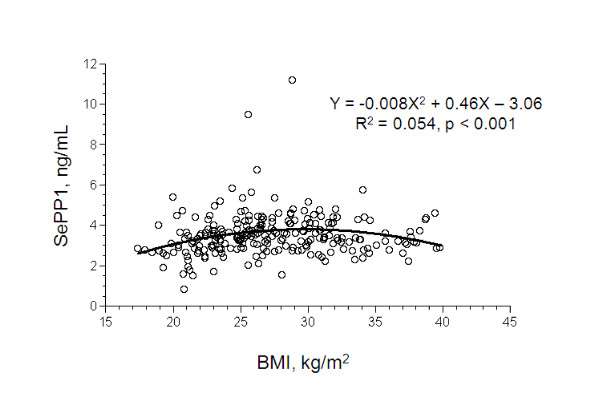
**Relationship of serum SEPP1 level and body mass index (BMI)**.

Serum SEPP1, but not GPX3 activity or total plasma Se, showed a significant quadratic relationship with body mass index (BMI) (r^2 ^= 0.054, P < 0.002), being lower in individuals at the low and high ends of the BMI range (figure 4). In addition to the regression analysis shown in figure 4, ANOVA, Tukey contrast and orthogonal contrast tests were performed. There were only 2 subjects with BMI < 18; these were included in the BMI < 25 group. The overall ANOVA was highly significant, p = 0.0012. By Tukey contrast tests comparing the means of the 3 groups, the mean of the lowest BMI group was highly significantly less than the mean of the overweight group, p = 0.0008. An orthogonal contrast test showed a significant quadratic trend in the means of the 3 groups, p = 0.003. The back-transformed SEPP1 means (± 1 S.D.) are as follows: BMI < 25: 3.17 (3.07-3.26); BMI 25-30: 3.69 (3.59-3.80); BMI > 30: 3.46 (3.36-3.58). Figure 4 makes evident the presence of two outlier subjects. With all values included in the quadratic regression of SEPP1 vs BMI, R^2 ^= 0.054, p = 0.0016. Excluding the two high values improved the fit slightly: R^2 ^= 0.0596, p = 0.0009.

### Determinants of Se Status

**Figure 5:**
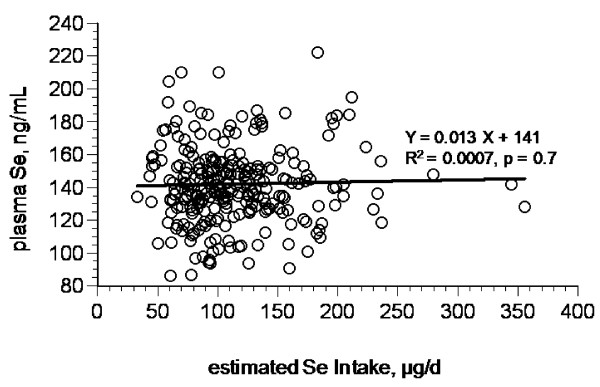
**Relationship of plasma Se level and estimated Se dietary intake**.

Plasma Se level was not significantly associated with Se intake estimated by FFQ (figure 5); however, it was significantly associated with numbers of servings of foods in four food groups: fish (r = 0.14, P < 0.022), fish subcategory "other" (including fish other than tuna; r = 0.15, P < 0.014); dairy (r = 0.13, P < 0.036), and cured meat (including sausage and luncheon meats; r = 0.15, P < 0.019). A discriminate analysis showed that food Se intake correctly predicted the plasma Se quartile for about 73% of the individuals. The foods selected by the analysis are shown in Table 3. Plasma Se level was not significantly different between men and women, or between users and non-users of nutritional supplements. We assumed that the maximum amount of Se consumed from supplements (<50 ug/day) would fall within normal day-to-day interindividual variations in Se-intake. That assumption is borne out by our finding of no significant differences in the plasma Se levels between supplement users and non-users for plasma Se. Plasma Se was not significantly associated with age, metabolic body weight (kg^0.75^), or serum concentration of folate, vitamin B_12 _or hCys.

**Table 3:** Foods Selected as Predictive of Plasma Se

Foods	Mean Se Intake (μg/day)	
	
	**1**^**st **^**Quartile:**	**4**^**th **^**Quartile:**
Fish, halibut, cooked, dry heat	1.87 ± 2.83	3.29 ± 4.06

Alcoholic beverage, beer, regular	0.93 ± 1.71	0.42 ± 0.81

Egg, whole, cooked, scrambled	9.67 ± 12.4	6.33 ± 6.02

Seeds, sunflower seed kernels, oil roasted, salt added	1.37 ± 2.31	3.79 ± 8.44

Nuts, mixed nuts, oil roasted, with peanuts, salt added	3.18 ± 4.84	2.16 ± 3.78

Pork, fresh, shoulder, blade, roasts, separable lean only, cooked, roasted	3.98 ± 3.73	3.11 ± 3.29

Buccal cell Se was not significantly different for men vs. women; however, that of users of nutritional supplements (9.22 [5.89, 14.45] ^4 ^ng/mg protein, n = 81) was significantly greater (p < 0.02) than that of non-users (8.03 [5.50, 11.73]^4 ^ng/mg protein, n = 179). Buccal cell Se was significantly associated with age (r = 0.14, P < 0.02) and with serum hCys (r = 0.24. P < 0.0001) on exponential bases. It was not, however, associated with BMI, metabolic body size, or serum concentrations of folate or vitamin B_12_.

The urinary Se level of women (geometric mean [-1S.D., +1 S.D.]; 57.7 [42.3, 78.6] ng/mg creatinine) was significantly greater (p < 0.003) than that of men (51.8 [39.8, 67.5]^3 ^ng/mg creatinine); that of users of nutritional supplements (geometric mean [-1S.D., +1 S.D.]; 59.3 [44.0, 80.0] ng/mg creatinine, n = 80) was significantly greater (p < 0.01) than that of non-users (53.5 [40.0, 71.5] ^3 ^ng/mg creatinine, n = 180). Urinary Se was not significantly associated with age or BMI; but it was significantly associated with metabolic body weight (kg^0.75^) (r = -0.14, p < 0.024) and with serum levels of folate (r = 0.17, P < 0.006) and vitamin B_12 _(r = 0.34, P < 0.0001) and negatively with serum hCys (r = -0.16, P < 0.009) on exponential bases.

This cohort showed heterogeneity with respect to the genotypes for each of the selenoproteins determined, three of which were significantly related to a biomarker of Se status (Table 4). Individuals with the *GPX1 *679 T/T genotype (rs1050450) constituted 11% of the cohort and had significantly lower (by 7%) plasma Se levels than those with the most prevalent (47%) *GPX1 *679 C/T genotype. Individuals with the *SEPP1 *25191 G/A genotype (rs3877899) constituted 44% of the cohort and had significantly lower (by 11%) serum SEPP1 levels than those with the *SEPP1 *25191 G/G genotype, which constituted a similar portion of the cohort. Individuals with the *SEP15 *811 T/C genotype (rs5845) constituted 31% of the cohort and had significantly lower (by 15%) buccal cell Se levels than those with the most prevalent (65%) *SEP15 *811 C/C genotype. No other differences were found in the values of Se biomarkers due to these genotypes, or due to GPX4 genotype (rs713041).

**Table 4:** Effects of selenoprotein genotype of biomarkers of Se status^1^.

gene	allele position-base	function/protein position	n	plasma Se ng/ml	GPX3 nmol/min/mg	serum SEPP1 ng/ml	buccal cell Se ng/mg prot	urine Se ng/mg creatinine
*GPX1*	679 T/T	Leu198Leu	28	135.7 ± 19.0^a^	3.67 ± 0.40	3.34 (2.58, 4.33)^2^	7.72 (5.10, 11.66)^2^	55.84 (42.80, 72.85)^2^
	679 T/C	Leu198Pro	113	139.5 ± 23.1^a,b^	3.60 ± 0.53	3.60 (2.86, 4.54)	8.86 (5.89, 13.31)	56.69 (41.85, 76.78)
	679 C/C	Pro198Leu	119	145.9 ± 24.3^b^	3.67 ± 0.58	3.30 (2.43, 4.49)	8.13 (5.44, 12.15)	53.74 (39.92, 72.35)

*GPX4*	718-C/C	UTR-3	74	142.0 ± 23.2	3.63 ± 0.53	3.40 (2.65, 4.38)	8.25 (5.34, 12.73)	56.86 (42.31, 76.43)
	718-C/T	UTR-3	135	141.3 ± 22.8	3.66 ± 0.56	3.43 (2.55, 4.62)	8.53 (5.86, 12.41)	54.78 (41.71, 71.96)
	718-T/T	UTR-3	51	143.9 ± 25.9	3.61 ± 0.53	3.49 (2.75, 4.42)	8.24 (5.25, 12.95)	54.14 (37.88, 77.38)

*SEPP1*	24731-A/A	Thr234Thr	11	129.7 ± 27.9	3.66 ± 0.49	4.07 (3.55, 4.66)	6.92 (4.64, 10.31)	59.01 (46.36, 75.12)
	24731-G/A	Thr234Ala	98	140.1 ± 23.0	3.57 ± 0.56	3.41 (2.55, 4.55)	8.41 (5.46, 12.95)	53.14 (40.52, 69.69)
	24731-G/G	Ala234Ala	151	144.2 ± 23.3	3.69 ± 0.53	3.41 (2.61, 4.45)	8.49 (5.75, 12.55)	56.38 (41.15, 77.23)
	25191-A/A	UTR-3	31	145.0 ± 28.4	3.76 ± 0.63	3.49 (2.91, 4.18)^ab^	8.88 (5.70, 13.84)	55.17 (39.58, 76.90)
	25191-G/A	UTR-3	115	141.8 ± 21.6	3.63 ± 0.58	3.24 (2.44, 4.31)^a^	8.55 (5.71, 12.81)	54.40 (39.95, 74.08)
	25191-G/G	UTR-3	114	141.5 ± 24.0	3.63 ± 0.47	3.62 (2.75, 4.76)^b^	8.10 (5.42, 12.09)	56.10 (42.58, 73.92)

*SEP15*	811-C/C	UTR-3	169	143.2 ± 24.4	3.63 ± 0.55	3.45 (2.72, 4.38)	8.77 (5.85, 13.14)^a^	56.73 (41.87, 76.87)
	811-T/C	UTR-3	81	139.0 ± 21.6	3.67 ± 0.53	3.38 (2.40, 4.74)	7.65 (5.06, 11.57)^b^	52.45 (39.32, 69.97)
	811-T/T	UTR-3	10	145.2 ± 23.1	3.56 ± 0.43	3.67 (3.10, 4.35)	8.27 (6.16, 11.10)^ab^	53.61 (44.36, 64.80)

^1 ^155 women, 106 men

^2 ^geometric mean (-1SD, +1SD)

^3 ^Values within a gene-biomarker group having the same lettered superscript were not significantly different (p > 0.05)

## Discussion

The available biomarkers of Se status have been developed for use in individuals suboptimally nourished with respect to the element. Indeed, they have been found generally useful in such cases, as differences in their values tend to correlate with differences in Se intake. More importantly, two of those biomarkers have direct functional significance: GPX3 participates in antioxidant function; while SEPP1 is involved in Se transport and also has antioxidant properties. However, these biomarkers are not informative of Se status under conditions of Se intake exceeding the level necessary to support their maximal expression, which coincides with the level apparently effective in reducing cancer risk.

This study demonstrates the problem of characterizing the Se status of a cohort that is not deficient in the element. It shows the discordance of biomarkers of Se status in a cohort of healthy Americans and the lack of utility of biomarkers with functional significance to assess Se status in a Se-adequate population. The most commonly used biomarker of Se status, plasma Se content, is presumed to reflect both the amounts of Se in various body pools and the level of Se intake [19]. At 142.0 ± 23.5 ng/ml, it showed that this cohort was of relatively high Se status. Half of subjects had plasma Se levels comparable to those of the upper quintile of the NHANES 2003-2004 cohort [11], and 6% had plasma Se levels in the range achieved only by Se-supplementation in the NPC trial [7]. No subject had a plasma Se level <70 ng/ml, above which Nève [12] noted subjects do not show GPX3 responses to Se-supplementation. Accordingly, plasma Se levels, all of which were above that threshold, were not associated with differences in GPX3 activity or SEPP1 level. This indicates maximal expression of those selenoproteins, rendering their measurement uninformative as biomarkers of Se status in this cohort. Differences in SeMet intake affect only the non-specific component of plasma Se, is also likely applicable to other such populations of relatively high Se status

Hill et al [13] estimated that GPX3 and SEPP1, when maximally expressed, should account for about 80 ng/ml of the Se in human plasma. Their estimate was based on the amount of Se associated with SEPP1 in one apparently Se-adequate individual, and the amount of Se reported by Avissar et al [11] to be associated with GPX3. The GPX3 activities and SEPP1 levels of our cohort indicate a slightly lower value, approximately 73 ng/ml, which may reflect weaknesses in our assumptions, particularly those concerning SEPP1. From the reports of Steinbrenner et al [36] and Méplan et al [37], we assumed the presence of multiple SEPP1 variants with an average molecular weight of 60 kD. We also assumed that SEPP1 contained an average of 9.9 g-atoms Se as SeCys per mole based on the findings of Saito et al [35] with human SEPP1; although the genetic coding of the protein would suggest the possibility of as many as 10 SeCys residues per mole [38]. Our estimate suggests that the amount of Se present as SeCys in GPX3 and SEPP1 comprised about 54% of the total amount of Se in the plasma of these non-deficient subjects, a lower percentage than observed (80%) by Deagen et al [39] for Se-deficient men in China. The differences between these different cohorts may reflect the preferential response of the non-specific plasma pool to SeMet. This phenomenon is indicated by nearly half (47%) of plasma Se occurring in the non-specific fraction in the present cohort. Indeed, the variation we observed in plasma Se level was almost exclusively limited to variation in the non-specific component. That this non-specific fraction was not confounded by such variables such as age, gender, metabolic body weight or methylation status (serum folate, vitamin B_12_, Hcy) suggests that it may be a useful biomarker of Se status in non-deficient populations; since differences in SeMet intake affect only the non-specific component of plasma Se, these findings are also likely applicable to other such populations of relatively high Se status.

We are not aware of buccal cells, which offer the advantage of sampling a metabolically active tissue, having been used previously to assess somatic cell Se. We found buccal cells to contain appreciable amounts of Se; however, their Se showed no significant correlation with plasma Se level. The levels of Se in buccal cells and urine were each positively associated with the use of nutritional supplements. In this regard, these biomarkers may be more useful than plasma Se, which did not show such an effect.

Urinary Se, which consists mostly of methylated selenosugars [40-42], was the only Se biomarker not in dynamic equilibrium with other pools of Se in the body, although it would be expected to show first-order relationships with such pools. That urinary Se was also positively associated with serum folate and vitamin B_12_, but negatively associated with serum hCys, is consistent with its metabolic production being dependent on the availability of methyl groups. This is supported by the findings of Gonzalez et al. [43] that serum Se level was positively associated with serum folate level and negatively associated with serum hCys level, the latter explaining nearly 6% of the variance of serum Se. The lack of a relationship between plasma Se and urinary Se suggests that these two pools are not in a first-order relationship. Because the non-specific Se was the only variable component of plasma Se, it's apparent that very little of that protein-bound pool turns over into urine. The dimorphic Se excretion between men and women adds to the list of sex-specific differences in (hepatic) Se metabolism, which has been observed in both rodents and humans [44].

The relationship of serum SEPP1 and BMI (r^2 ^= 0.054; p < 0.001), with greatest values among subjects with BMIs of 25-30 (figure 4), was unexpected as Kimmons et al [45] noted low plasma Se levels (<100 ng/ml) somewhat more frequently among women in this BMI class in the NHANES III cohort. That this may reflect the dysregulation of gluconeogenesis in obesity is suggested by studies in cultured cells that have shown SEPP1 to be regulated as a gluconeogenic enzyme [46-48].

It is also clear that genetic variability contributes to variance of Se biomarkers. Of the four allelic selenoprotein variants studied three, *GPX1, SEPP1 *and *SEP15*, were significantly related to the values of a Se biomarker (Table 4). That individuals with *GPX1 *679T/T alleles showed significantly lower plasma Se levels than those of the C/C alleles is of particular interest, as the former genotype has been associated with increased risk to cancers of the lung [49] and breast [50]. That some SEPP1 genotypes have less (11%) SEPP1 expression than others, and that low BMI individuals had significantly lower SEPP1 than those with mid-level and high BMI, it is possible that SEPP1 in some individuals in this cohort may not be maximally expressed, even at these apparently adequate levels of Se intake.

The estimated intakes of macronutrients were similar to those reported for other cohorts of Americans [24]. However, estimated Se intake did not significantly correlate with any Se-biomarker. This is not surprising, considering the inherent errors in determining nutrient intakes from an FFQ and in estimating the amount of Se in particular foods, which can vary considerably depending on the location and/or means of food production [51]. The results of the FFQ method suggested that a core group of 22 foods provided 80% of the Se consumed by this cohort, a number similar to that estimated by Schubert et al [23] for the American population. This core included pork, beef and wheat products, the Se contents of which Finley et al [52,53] showed can vary enormously (by 3-, 11- and 57-fold, respectively, for items purchased in the upper Midwest). Such great uncertainty severely compromises the value of Se intake estimated in this way as a useful indicator of Se status.

## Conclusions

The assessment of Se status in individuals that are not deficient in Se calls for the use of non-classical/unconventional parameters that may be informative despite their limited direct functional significance. In a cohort of healthy Americans, Se intake, estimated using accepted FFQ methodology, was not associated with any biomarkers of Se status; the high degree of uncertainty inherent in this approach rendered it unsuitable for predicting Se status. Two widely used Se biomarkers, GPX3 and SEPP1, showed little correlation with one another. Each appeared to be maximally expressed, neither being associated with total plasma Se. In contrast, Se in the non-specific component of plasma was positively associated with total plasma Se.

It is clear that plasma Se comprises the Se specifically incorporated as SeCys in GPX3 and SEPP1, as well as Se present as SeMet incorporated non-specifically into the plasma proteins. One would also expect plasma also to contain small amounts of Se-metabolites some of which may be noncovalently associated with plasma proteins. In this cohort the non-specific pool was the dominant component, comprising 47% of plasma Se and representing most of the variance in that biomaker. It would therefore appear to be the most useful parameter of Se status in such a cohort.

What we have called the non-specific component of plasma Se was imputed from our measurements of other biomarkers. In order to use it for assessing Se status in non-deficient individuals, it will be necessary to assess it directly. This will mean developing means to assess its various components, which we would expect to include mostly protein-bound SeMet, but also smaller amounts of Se bound to protein-thiols and non-protein bound Se-metabolites including selenosugars.

These results demonstrate that factors other than Se intake that can contribute to variance in Se biomarkers used to assess Se status. Such factors include genotype, methyl-group status, and BMI. It is possible that these factors may contribute to heterogeneity in biomarker responses to Se-supplementation, a problem noted by Ashton et al [54].

## Competing interests

The authors declare that they have no competing interests.

## Authors' contributions

The authors' responsibilities were as follows: GFC, JCW, CDD and JAM designed the research; GFC, JDW, MIJ, HZ, AJS, EOU, LS, AH and CSH conducted the research; LKJ analyzed the data; GFC and MIJ wrote the paper and had responsibility for its final content.

The authors read and approved the manuscript.
